# Prognostic performance of pretreatment systemic immune-inflammation index in women with epithelial ovarian cancer

**DOI:** 10.2144/fsoa-2023-0108

**Published:** 2023-08-28

**Authors:** Kehinde S Okunade, Sarah O John-Olabode, Adaiah P Soibi-Harry, Austin C Okoro, Adebola A Adejimi, Iyabo Y Ademuyiwa, Benedetto Osunwusi, Hameed Adelabu, Omolola Salako

**Affiliations:** 1 Department of Obstetrics & Gynaecology, College of Medicine, University of Lagos, Surulere, Lagos, Nigeria; 2 Department of Obstetrics & Gynaecology, Lagos University Teaching Hospital, Surulere, Lagos, Nigeria; 3 Center for Clinical Trial, Research, & Implementation Science (CCTRIS), College of Medicine, University of Lagos, Lagos, Nigeria; 4 Department of Haematology & Blood Transfusion, College of Medicine, University of Lagos, Lagos, Nigeria; 5 Department of Community Health & Primary Care, College of Medicine, University of Lagos, Lagos, Nigeria; 6 Department of Nursing Science, College of Medicine, University of Lagos, Lagos, Nigeria; 7 Department of Radiation Biology, Radiotherapy & Radiodiagnosis, College of Medicine, University of Lagos, Lagos, Nigeria

**Keywords:** Africa, EOC, ovarian cancer, overall survival, prognosis, progression-free survival, SII

## Abstract

**Purpose:**

This study investigated the prognostic performance of the systemic immune-inflammation index (SII) in patients with epithelial ovarian cancer (EOC) in Lagos, Nigeria.

**Methods:**

We performed a secondary analysis of the data of 91 women who had treatment for EOC between 2009 and 2018. The associations between pretreatment SII and survivals were tested.

**Results:**

Pretreatment SII more than 610.2 was a significant independent predictor of reduced progression-free survival (HR = 2.68; 95% CI, 1.17 to 6.09) while SII greater than 649.0 was a significant independent predictor of reduced 3-year overall survival (HR = 2.01; 95% CI, 1.01 to 3.99).

**Conclusion:**

These findings suggest that high SII may be a potential prognostic indicator and useful marker for more intensive surveillance and design of personalized treatment in patients with EOC.

## Plain language summary

This study looked at how the systemic immune-inflammation index (SII) can predict the outcomes of patients with epithelial ovarian cancer (EOC). To do this, the data of 91 women who received treatment for EOC between 2009 and 2018 were analyzed. The study concluded that when the SII level was higher than 610.2 and 649.0, it was linked to a higher likelihood of EOC progressing sooner and of reduced survival at the 3-year mark, respectively. This suggests that a high SII might be a useful predictor to understand how EOC could progress and how well patients with EOC might survive.

Ovarian cancer is the eighth most common cancer in women worldwide [1] and the second most common gynecologic cancer in Nigeria [2]. The epithelial histotypes (EOC) account for more than 90% of all ovarian cancer [1,3] with more than 70% of these diagnosed at a later stage of the disease [4] when it is characterized by poor survival prognosis [5]. A risk-stratification strategy using patient- and tumor-specific characteristics is now being proposed to predict survival and to identify EOC patients who require more intensive treatment and individualized follow-up surveillance plans [6]. This is especially more important in resource-limited settings such as Nigeria with much higher mortality seen as patients advance through the disease trajectory [4].

Several clinicopathological factors, including histology, tumor stage, residual disease after surgical debulking and response to chemotherapy have been proposed to predict EOC outcomes [5,7]. However, there is currently conflicting evidence on the reliability of these factors to accurately predict survival when used before complete primary treatment outcomes in patients [5,8]. In the past few decades, the links between inflammation and cancer development have gained significant interest [9]. Increasing evidence suggests that the activation of inflammation plays a crucial role in cancer metastasis and recurrence [10,11]. There is, therefore, a growing interest in the role of systemic inflammatory response markers such as neutrophil-lymphocyte ratio (NLR), platelet-lymphocyte ratio (PLR) and systemic immune-inflammation index (SII) as predictors of survival in several malignancies including ovarian cancer [12–17]. The use of these basic and cheap hematologic indices as reliable prognostic markers is novel and necessary for the identification of high-risk patients who may benefit from maintenance therapy after completion of their primary treatment with primary debulking surgery and adjuvant chemotherapy or neoadjuvant chemotherapy, interval debulking surgery and adjuvant chemotherapy [16,18].

The NLR is the most extensively utilized systemic inflammatory response marker to assess the potential balance between neutrophil-dependent pro-tumor inflammation and lymphocyte-associated anti-tumor immune response [16]. However, SII integrates the combination of neutrophil, platelet and lymphocyte counts as inflammatory markers. Neutrophils promote the proliferation and metastasis of tumor cells, aid the undermining of immune surveillance, repair the senescent cancer cell and suppress T cell activation to promote immune evasion [19]. Platelets protect circulating tumor cells from shearing forces in arteries, allowing them to shift from epithelial to mesenchymal state [4] while lymphocytes demonstrate anti-tumor activities of mediating adaptive and non-adaptive immune responses by recognizing foreign antigenic determinants expressed on tumor cells and mounting an effective, tumor-specific immune response toward these determinants [20].

Few documented studies have examined the prognostic significance of SII in EOC but none of these studies was conducted among black African women treated for EOC. This study, therefore, investigated the prognostic value of pretreatment peripheral blood SII in patients with EOC managed at the Lagos University Teaching Hospital, Lagos, Nigeria during a 10-year review period.

## Materials & methods

### Study design

This was a secondary analysis of datasets of the retrospective cohort study by John-Olabode *et al.* in 2021 [16] on the prognostic role of pretreatment neutrophil-to-lymphocyte ratio in epithelial ovarian cancer at the Lagos University Teaching Hospital, Lagos, Nigeria over 10 years (March 2009–February 2018). The hospital is the teaching hospital of the College of Medicine, University of Lagos that is located on the mainland of Lagos. The hospital acts mainly as a referral center for other government-owned and private hospitals in Lagos and its surrounding States [16].

### Eligibility criteria

We analyzed the data of 91 women diagnosed with epithelial ovarian cancer (EOC) who had complete primary treatment with either primary debulking surgery and adjuvant chemotherapy or neoadjuvant chemotherapy, interval debulking surgery and adjuvant chemotherapy and who had sufficient clinical records (including the SII and survival data) in the dataset. Adjuvant chemotherapy protocols comprised six or three cycles every three weeks of paclitaxel, 175 mg/m^2^ intravenously over 3 h followed by carboplatin, area under the curve (AUC) 5–6 intravenously over 30–60 min on day one following upfront treatment with either primary debulking surgery or neoadjuvant chemotherapy (three to four chemotherapy cycles) and interval debulking surgery respectively [18]. Exclusion criteria included patients with non-epithelial ovarian cancer; those with Eastern Cooperative Oncology Group (ECOG) performance status of 2–4 [21]; those with active infection or hematologic disease or medication with an immunosuppressive agent; and those with failure to complete primary treatment. Variables extracted for analyses included the patient's age, parity, menopausal status, BMI, serum CA-125 concentration, co-existing morbidity (hypertension, diabetes mellitus, cardiac, kidney and liver disease), complete blood count, type of upfront treatment, surgical debulking status [22,23], presence of ascites, International Federation of Gynecology and Obstetrics (FIGO) stage [24], histological subtype [25], progression-free survival and overall survival. Residual disease after surgery (primary or interval debulking) was reported as optimal or suboptimal resection. Optimal resection was defined as a combination of no macroscopic residual disease (R0) or residual disease with a maximal diameter of <1 cm (R1) while suboptimal resection was defined as macroscopic residual disease >1 cm in maximum diameter (R2) [26]. We also extracted the pretreatment peripheral blood neutrophils, platelets and lymphocytes values. We calculated the systemic immune-inflammation index (SII) by dividing the product of neutrophil (N) and platelet counts (P) by the lymphocyte counts (L) = (N × P)/L.

### Study outcomes

We assessed two study outcomes: progression-free survival (PFS), defined as the time interval between completion of primary treatment and the first evidence of disease progression as assessed by clinical examination, elevated tumor marker (serum CA125 levels) and/or radiological studies; and overall survival (OS), defined as the time interval between completion of primary treatment and death from all causes or last follow-up since completion of treatment for patients who were still alive. Survival data were censored after 3 years of patient follow-ups.

### Statistical analysis

Data were analyzed using the SPSS version 28.0 statistical package for Windows (manufactured by IBM Corp., NY, USA) and descriptive statistics were computed for the patients' baseline characteristics. Characteristics classified as continuous variables were described using mean and standard deviation (if normally distributed) and median and interquartile range (if skewed). Categorical variables were described using frequencies and percentages. The Receiver operating characteristic (ROC) curve analysis with Youden's index was used to estimate the optimum cut-off values for the SII in predicting the PFS and 3-year OS. The Kaplan–Meier curve analysis with the Log Rank (Mantel-Cox) test statistic [27] was used to test the association between SII stratified by their cut-off values and survival (PFS and OS). We censored patients without tumor recurrence or those alive at the last follow-up. We determined the Hazard Ratios (HR) Using the multivariate Cox regression models with adjustments made for other possible confounding factors. The final multivariate models were built to include variables with a p-value < 0.2 in the bivariate analyses. Statistical significance in the multivariate model was reported at p-value < 0.05.

### Ethical considerations

Approval for this study was granted by the Health Research Ethics Committee of the Lagos University Teaching Hospital (ADM/DCST/HREC/APP/3699) before accessing the patients' datasets for analysis. The study was conducted in compliance with the ethical standards of the Lagos University Teaching Hospital on human subjects as well as with the World Medical Association principles of the Declaration of Helsinki.

## Results

The datasets of 91 of the 155 cases of ovarian cancer managed in the hospital during the 10-year review period in the primary study [16] were included in the data analyses. Excluded from the analyses were 29 women with non-epithelial ovarian cancer, 4 with incomplete hematologic parameters, 5 with an active infection or hematologic disease or on medication with an immunosuppressive agent, 5 with ECOG suboptimal performance status of two to four, 13 who were unable to complete primary treatment, and 8 with missing survival data.

The patients' mean age at presentation was 47.6 ± 14.0 years. The major proportions of the patients were diagnosed with FIGO stage III and IV disease (n = 63, 69.2%), and high-grade serous carcinomas (n = 59, 64.8%). A higher proportion of these patients, n = 57 (62.6%) had primary debulking surgery rather than neoadjuvant chemotherapy as their upfront primary treatment. The detailed baseline characteristics of the patients are highlighted in Table 1.

**Table 1.** Baseline characteristics of patients with epithelial ovarian cancer (n = 91).

Characteristics	n (%)
Mean age (±SD) in years	47.6 ± 14.0
Mean BMI (±SD) in kg/m^2^	23.6 ± 5.1
Median serum CA-125 levels (IQR) in U/ml	113.6 (48.8, 597.0)
Median SII (IQR)	576.4 (295.6, 1218.7)
Parity	
Nulliparous	41 (45.1)
Multiparous	50 (54.9)
Menopausal status	
Premenopause	51 (56.0)
Postmenopause	40 (44.0)
Comorbidity	
Yes	16 (17.6)
No	75 (82.4)
Upfront primary treatment	
PDS	57 (62.6)
NACT and IDS	34 (37.4)
Ascites	
Yes	36 (39.6)
No	55 (60.4)
FIGO stage	
Early (I and II)	28 (30.8)
Advanced (III and IV)	63 (69.2)
Residual disease status	
Optimal (R0/R1)	39 (42.9)
Suboptimal (R2)	52 (57.1)
Histologic subtype	
Type I (LGSC and others)	32 (35.2)
Type II (HGSC)	59 (64.8)

CA: Cancer antigen; FIGO: International Federation of Gynecology and Obstetrics; HGSC: High-grade serous carcinoma; IDS: Interval debulking surgery; IQR: Interquartile range; LGSC: Low-grade serous carcinoma; NACT: Neoadjuvant chemotherapy; PDS: Primary debulking surgery; SD: Standard deviation; SII: Systemic immune-inflammation index.

**Figure 1.**
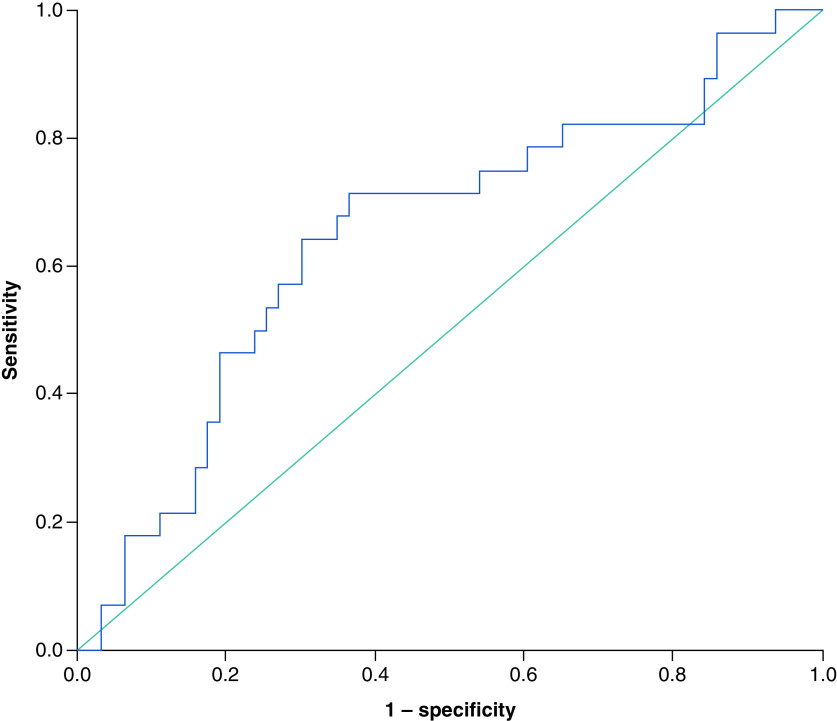
The receiver operating characteristic curve for progression-free survival. Showing an area under the curve of 0.65 (95% CI: 0.52–0.78), a sensitivity of 71.4% and a specificity of 61.9%.

**Figure 2.**
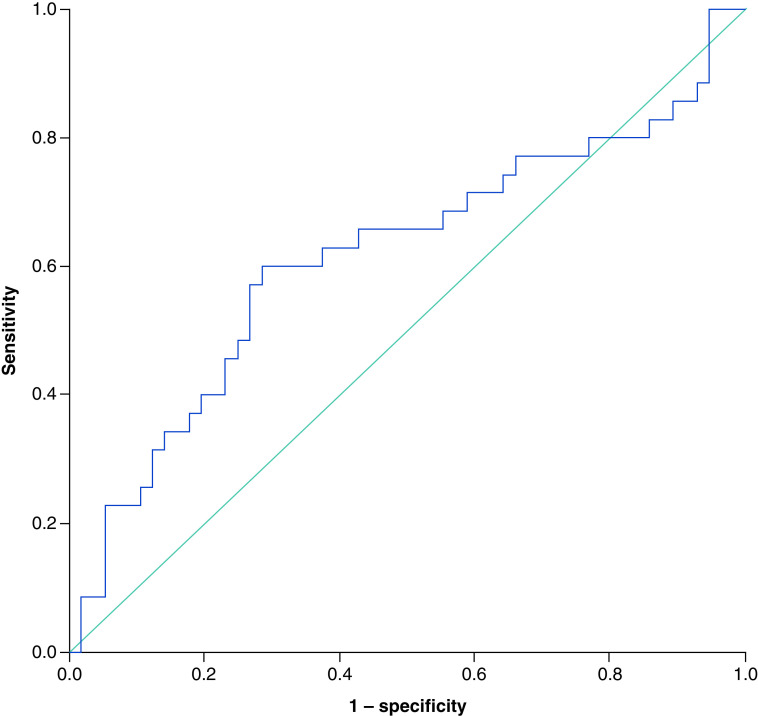
The receiver operating characteristic curve for 3-year overall survival. Showing an area under the curve of 0.62 (95% CI: 0.50–0.74), a sensitivity of 60.0% and a specificity of 69.6%

The optimal SII cut-off value for PFS was 610.2 × 10^9^ cells/l with an area under the curve (AUC) of 0.65 (95% CI: 0.52–0.78), the sensitivity of 71.4% and specificity of 61.9% [Figure 1]. For 3-year OS, the optimal SII cut-off value was 649.0 × 10^9^ cells/l with an area under the curve (AUC) of 0.62 (95% CI: 0.50–0.74), a sensitivity of 60.0% and specificity of 69.6% [Figure 2].

**Figure 3.**
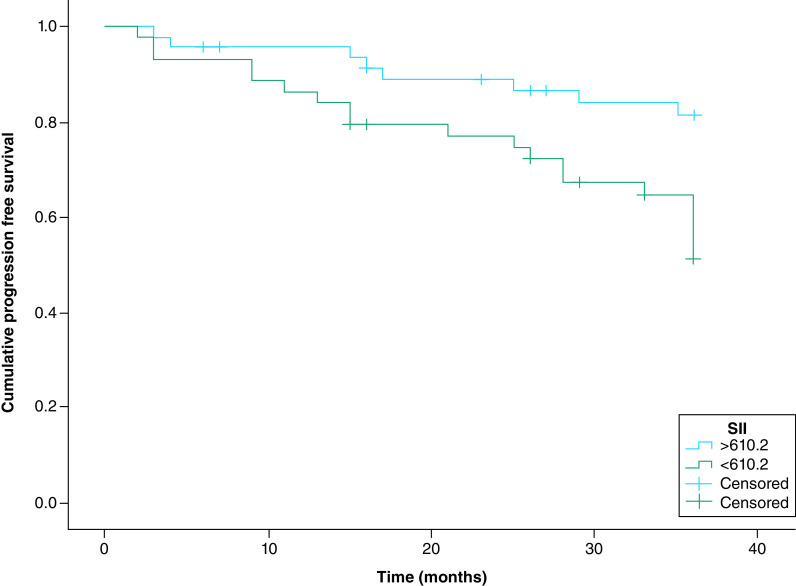
The Kaplan–Meier survival curve of progression-free survival. Stratified by the SII cut-off values – SII >610.2 x 10^9^ cells/L was significantly associated with a shorter progression-free survival in epithelial ovarian cancer patients (p = 0.006).

**Figure 4.**
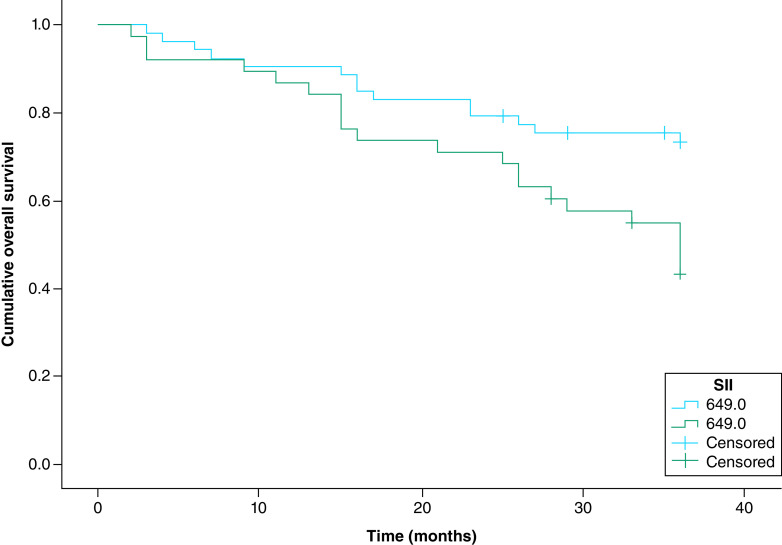
The Kaplan–Meier survival curve of 3-year overall survival. Stratified by the SII cut-off values – SII >649.2 x 10^9^ cells/l was significantly associated with a shorter overall survival in epithelial ovarian cancer patients (p = 0.009).

The Kaplan–Meier (KP) survival curve stratified by the SII cut-off values of 610.2 × 10^9^ cells/l for PFS and 649.0 × 10^9^ cells/l for 3-year OS showed that the PFS (p = 0.006) and OS (p = 0.009) were significantly shorter in EOC patients with SII values above the optimal cut-offs than in those with SII below the cut-offs [Figures 3 & 4].

Following adjustments for parity, serum CA-125 levels, comorbidity and FIGO stage in the multivariate Cox regression models, pretreatment SII more than 610.2 × 10^9^ cells/l was a significant independent predictor of reduced PFS (hazard ratio [HR] = 2.68; 95% CI, 1.17 to 6.09, p = 0.020) (Table 2). On adjusting for parity, pretreatment SII more than 649.0 × 10^9^ cells/l was a significant independent predictor of reduced 3-year OS (HR: 2.01, 95% CI: 1.01–3.99, p = 0.046) (Table 3).

**Table 2.** Bivariate and multivariate analyses for progression-free survival.

Characteristics	Category	Univariate	Multivariate	
		p-value	HR (95% CI)	p-value
Age	≥48 vs <48 years	0.934	–	–
Parity	Multiparous vs nulliparous	0.061	1.89 (0.82–4.31)	0.131
Menopausal status	Postmenopause vs premenopause	0.876	–	–
BMI	≥24.0 vs <24.0 kg/m^2^	0.619	–	–
Serum CA-125 levels	≥114.0 vs <114.0 U/ml	0.094	1.42 (0.51–3.97)	0.503
Comorbidity	Yes vs No	0.059	2.88 (1.20–6.89)	0.018
Upfront primary treatment	NACT and IDS vs PDS	0.743	–	–
Ascites	Yes vs No	0.593	–	–
FIGO stage	Advanced vs early	0.130	1.35 (0.39–4.64)	0.635
Residual disease status	Optimal vs suboptimal	0.313	–	–
Histologic subtype	Type II vs Type I	0.942	–	–
Pretreatment SII	≥610.2 vs <610.2 × 10^9^ cells/l	0.007	2.68 (1.17–6.09)	0.020

BMI: Body mass index; CA: Cancer antigen; FIGO: International Federation of Gynecology and Obstetrics; HR: Hazard ratio; IDS: Interval debulking surgery; NACT: Neoadjuvant chemotherapy; PDS: Primary debulking surgery; Type I includes endometrioid carcinoma, clear cell carcinoma, mucinous carcinoma, and low-grade serous carcinomas; Type II includes high-grade serous carcinomas; SII: Systemic immune-inflammation index.

**Table 3.** Bivariate and multivariate analyses for overall survival.

Characteristics	Category	Univariate	Multivariate	
		p-value	HR (95% CI)	p-value
Age	≥48 vs <48 years	0.361	–	–
Parity	Multiparous vs nulliparous	0.029	1.96 (0.95–4.05)	0.069
Menopausal status	Postmenopause vs premenopause	0.800	–	–
BMI	≥24.0 vs <24.0 kg/m^2^	0.664	–	–
Serum CA-125 levels	≥114.0 vs <114.0 U/ml	0.548	–	–
Comorbidity	Yes vs No	0.742	–	–
Upfront primary treatment	NACT and IDS vs PDS	0.657	–	–
Ascites	Yes vs no	0.359	–	–
FIGO stage	Advanced vs early	0.506	–	–
Residual disease status	Optimal vs suboptimal	0.947	–	–
Histologic subtype	Type II vs Type I	0.574	–	–
Pretreatment NLR	≥649.0 vs <649.0 × 10^9^ cells/l	0.019	2.01 (1.01–3.99)	0.046

BMI: Body mass index; CA: Cancer antigen; FIGO: International Federation of Gynecology and Obstetrics; HR: Hazard ratio; IDS: Interval debulking surgery; NACT: Neoadjuvant chemotherapy; PDS: Primary debulking surgery; Type I includes endometrioid carcinoma, clear cell carcinoma, mucinous carcinoma, and low-grade serous carcinomas; Type II includes high-grade serous carcinomas; SII: Systemic immune-inflammation index.

## Discussion

The search for the most reliable pretreatment prognostic systemic inflammatory response markers continues. Systemic immune-inflammation index (SII), an inflammation- and immunity-related biomarker, defined as the ratio of peripheral blood neutrophils and platelets to lymphocytes is currently gaining prominence. In this secondary analysis of EOC patients managed over a 10-year review period [16], pretreatment SII >610.2 × 10^9^ cells/l was recorded as a significant independent predictor of reduced PFS while SII >649.0 × 10^9^ cells/l was a significant independent predictor of reduced 3-year OS.

Previous studies have selected SII cut-offs for stratification based on either the median values in the patients' cohorts or the ROC curve analysis for each of the survival outcomes [14,15,28,29]. It is still difficult to ascertain which of these methods should be recommended. In similarity to the study by Bizzarri *et al.* [28], we choose the SII value to stratify the EOC patients into low- and high-SII groups in this current study based on the ROC curve analysis. We selected optimal cut-offs of 610 and 649 for PFS and OS respectively in close similarity to the value of 612 used for both PFS and OS by Nie *et al.* [15] and the 600 for PFS by Wang *et al.* [28] but in wide variation to the much higher cut-off of 1000 selected by Bizzarri *et al.* [29] that included only patients with FIGO-stage I–II and IIIA1 EOC. Overall, these patterns suggest that a higher SII cut-off may be needed for predicting overall survival and early FIGO stage disease than the values required for predicting progression-free survival and more advanced stage disease. However, a more robust carefully designed longitudinal study will be necessary to further validate these findings.

Elevated SII reflects neutrophilia in the tumor microenvironment (supports cancer cell invasion, migration, and angiogenesis leading to the suppression of the antitumor immune reaction and cancer progression) [29], thrombocytosis (suggesting the highly reactive cellular mediators of immunity, primary hemostasis, and inflammation which plays important roles in cancer growth and metastasis) [30]; and lymphopenia (causing a weak and insufficient immunologic reaction to the tumor) [31]. The findings of SII as an independent predictor of both progression-free and overall survival in patients with EOC in this study corroborated that of the studies by Nie *et al.* in 2019 [15] and Wang *et al.* in 2022 [28]. Both studies [15,28] examined the impact of SII on survival in EOC patients who had neoadjuvant chemotherapy as their upfront treatment and reported shorter PFS and OS rates in the high SII group than in patients in the low SII group. These findings were further corroborated in a recent meta-analysis of six studies involving 1546 patients by Mao and Yang where a high SII significantly predicted poor OS and PFS in patients with ovarian cancer [32]. However, these findings are at variance with the published work by Farolfi *et al.* in 2018 [14] which reported that SII was not independently correlated with survival even after adjusting for all possible confounders. The conflicting findings in our study and others [15,28] and that of Farolfi *et al.* [14] may be attributed to the very high optimum cut-off of 730 selected to stratify SII into the high-SII and low-SII groups by Farolfi and colleagues [14] compared with the values ranging from 610 to 649 cut-offs used in this study and others [15,28].

The major limitations of this study, as similarly highlighted in the primary study [16], were the high number of EOC cases with incomplete clinical data required for analysis and its retrospective design, which may have led to bias in the data analysis. The single-institutional setting of the study may also make the generalization of the findings to other settings difficult. Finally, the number of EOC patients extracted from the datasets may be inadequate to power the study. However, as this topic has not attracted sufficient attention, our study will add to the growing body of literature. In addition, our study is the first, to our knowledge, that validated the prognostic impact of SII, as reported from similar studies in North America, Asia, and Europe, among black African women who had treatment for EOC.

## Conclusion

Our study reported that systemic immune-inflammation index (SII) at optimum cut-off values of 610.2 × 10^9^ cells/l and 649.0 × 10^9^ cells/l is an independent prognostic predictor of PFS and OS respectively in patients with EOC. These findings, therefore, suggest that high SII may be a potential prognostic indicator and useful marker for more intensive surveillance and design of personalized treatment in patients with EOC. However, a more robust, and long-term prospective multicenter study among black African women is required to further validate the findings of this study.

**Summary points**

- Increasing evidence suggests that the activation of inflammation plays a crucial role in cancer metastasis and recurrence.
- There is a growing interest in the role of systemic inflammatory response markers such as neutrophil-lymphocyte ratio, platelet–lymphocyte ratio and systemic immune-inflammation index as predictors of survival in several malignancies including ovarian cancer.
- Few studies have examined the prognostic significance of SII in epithelial ovarian cancer but none of these studies was conducted among black African women treated for epithelial ovarian cancer.
- Our study reported that pretreatment SII more than 610.2 was a significant independent predictor of reduced progression-free survival (95% CI, 1.17 to 6.09) while SII greater than 649.0 was a significant independent predictor of reduced 3-year OS (95% CI, 1.01 to 3.99).
- Our findings suggest that high SII may be a promising predictor and valuable prognostic indicator of disease progression and survival in epithelial ovarian cancer.
